# The role of ROS signaling in cross-tolerance: from model to crop

**DOI:** 10.3389/fpls.2014.00754

**Published:** 2014-12-23

**Authors:** Ilse Barrios Perez, Patrick J. Brown

**Affiliations:** Department of Crop Sciences, University of IllinoisUrbana, IL, USA

**Keywords:** systemic resistance, oxidative stress, GST, oxylipin, quantitative disease resistance, acclimation

## Abstract

Reactive oxygen species (ROS) are key signaling molecules produced in response to biotic and abiotic stresses that trigger a variety of plant defense responses. Cross-tolerance, the enhanced ability of a plant to tolerate multiple stresses, has been suggested to result partly from overlap between ROS signaling mechanisms. Cross-tolerance can manifest itself both as a positive genetic correlation between tolerance to different stresses (inherent cross-tolerance), and as the priming of systemic plant tolerance through previous exposure to another type of stress (induced cross-tolerance). Research in model organisms suggests that cross-tolerance could be used to benefit the agronomy and breeding of crop plants. However, research under field conditions has been scarce and critical issues including the timing, duration, and intensity of a stressor, as well as its interactions with other biotic and abiotic factors, remain to be addressed. Potential applications include the use of chemical stressors to screen for stress-resistant genotypes in breeding programs and the agronomic use of chemical inducers of plant defense for plant protection. Success of these applications will rely on improving our understanding of how ROS signals travel systemically and persist over time, and of how genetic correlations between resistance to ROS, biotic, and abiotic stresses are shaped by cooperative and antagonistic interactions within the underlying signaling pathways.

## INTRODUCTION

As sessile organisms, plants must adapt to adversity. Diverse biotic and abiotic stresses trigger systemic defense responses that involve the production of reactive oxygen species (ROS). This review discusses overlap between ROS-inducing stress responses as a possible explanation for two distinct phenomena: the enhancement of plant stress tolerance following previous exposure to another type of stress (induced cross-tolerance), and the ability of specific genetic variants to provide resistance to multiple distinct stresses (inherent cross-tolerance). Since ROS-triggered reactions mediate stresses that impact crop yield and quality, we also consider the gaps in the knowledge required to translate this research from model plants to crops.

## ROS HOMEOSTASIS AND LOCALIZATION

Reactive oxygen species are unstable molecules produced both as a result of normal aerobic metabolic processes inside the plant cell, and in response to abiotic stresses including drought, heat, high salinity, high light, osmotic stress, metal toxicity, and the presence of xenobiotics like herbicides and ozone (O_3_). ROS are also produced in response to biotic stresses during the ‘oxidative burst.’ ROS molecules include superoxide (O_2_^-^), hydrogen peroxide (H_2_O_2_), and singlet oxygen (O_2_*), and cause cell damage through lipid and protein oxidation and nucleic acid degradation (Apel and Hirt, 2004). The conflict between ROS toxicity and signaling roles has led to a tightly regulated equilibrium between ROS generation and scavenging. Oxidative stress in plant cells results from a disturbance in this equilibrium (Halliwell, 2006) and triggers both enzymatic and non-enzymatic mechanisms for scavenging free oxygen radicals. Non-enzymatic scavenging mechanisms involve the production of antioxidants including anthocyanins, ascorbate, carotenoids, and glutathione. Enzymatic scavenging mechanisms include the production of superoxide dismutase (SOD), ascorbate peroxidase (APX), and catalase (CAT; Apel and Hirt, 2004). Glutathione S-transferases (GSTs) and cytochrome P450s also scavenge ROS or their by-products.

Reactive oxygen species localization is specific to the type of stress to which the plant is subjected. Physical stresses that lower the rate of carbon fixation lead to photooxidative stress, which involves light-dependent ROS overproduction in the chloroplast. Such stresses include drought, high salinity, high temperature and xenobiotic compounds like methyl viologen (MV; “paraquat”). In contrast, O_3_ treatment, H_2_0_2_ treatment, and pathogen attack induce the accumulation of ROS in the apoplast. O_3_ is delivered directly into the extracellular space via stomates, where it is broken down into hydrogen peroxide and superoxide (Vainonen and Kangasjärvi, 2014). During the oxidative burst produced in response to pathogen attack, a class of NADPH oxidases in the cell membrane (respiratory burst oxidase homologues, or RBOHs) reduce oxygen into superoxide, which quickly forms hydrogen peroxide and diffuses from cell to cell through the extracellular space. Specific isozymes of APX and SOD localize to the cytosol, chloroplasts, apoplast, peroxisomes and mitochondria, whereas CAT localizes exclusively to peroxisomes. Lipid-soluble non-enzymatic antioxidants like anthocyanins and carotenoids are associated with cell and organelle membranes, while water soluble ascorbate and glutathione localize to the cytosol, chloroplasts, and other subcellular compartments (Mittler, 2002).

## ROS AS BASAL RESISTANCE SIGNALING MOLECULES LINKING BIOTIC AND ABIOTIC STRESS

### SENSING

It is not clear exactly how plant cells sense ROS, as specific ROS receptors have not yet been identified. One possibility is that ROS directly modify redox-sensitive proteins, including phosphatases (Apel and Hirt, 2004), and heat shock transcription factors (HSFs; Miller and Mittler, 2006). In *Arabidopsis*, overexpression of HSF-A1b conferred higher resistance to both drought and bacterial infection; this response was found to be dependent on H_2_O_2_ signaling but independent of hormone signaling, placing it within the basal immune system (Bechtold et al., 2013). Transcriptome analysis of rice responses to cold, heat and oxidative stress also suggests that HSFs play a crucial role in basal immunity. Binding sites for HSFs were significantly enriched in the promoters of upregulated genes in all three stress treatments, leading to the hypothesis that “the HSF/HSP regulon may be regarded as the central regulator of plant stress responses involving ROS accumulation” (Mittal et al., 2012).

### SIGNAL TRANSDUCTION

Zinc finger and WRKY transcription factors are both widely involved in the regulation of ROS-related defense genes. The expression of several zinc finger proteins in *Arabidopsis*, ZAT7 and ZAT12, is strongly upregulated by oxidative stress in APX knockouts and in response to H_2_0_2_ and MV treatment (Rizhsky, 2004). ZAT12 was later found to be involved in the regulation of resistance to high light, osmotic and oxidative stress, with overexpression mutants showing higher stress tolerance (Davletova et al., 2005). ZAT10 has a dual role as both inducer and repressor of ROS-responsive genes under salt, drought and osmotic stresses (Sakamoto et al., 2004; Mittler et al., 2006). ZAT6 positively regulates resistance to salt, drought, and chilling stress as well as resistance to bacterial infection by modulating ROS-level and SA-related gene expression (Shi et al., 2014). The WRKY transcription factor family includes several genes important for the disease response (Pandey and Somssich, 2009), and WRKY transcription factors participate in the cross-talk between biotic and abiotic resistance through ROS gene-related modulation (Mittler et al., 2011). WRKY25 is induced under heat, osmotic and oxidative stress, and appears to be regulated by ZAT12. Unlike ZAT12, however, its overexpression did not result in significantly higher tolerance (Rizhsky, 2004). WRKY70 is a key regulator of plant disease response and a known crossroads between the SA and JA pathways, being induced by the former and inhibited by the latter (Li et al., 2004), and interacts directly with ZAT7 (Ciftci-Yilmaz et al., 2007). WRKY30 is rapidly induced by inoculation with several pathogen-associated molecular patterns (PAMPs), and oxidative (MV) stress; its overexpression in young plants conferred resistance to oxidative and salt stress (Scarpeci et al., 2013, p. 30).

### SIGNAL AMPLIFICATION

Respiratory burst oxidase homologues (RBOHs) are a small family of membrane-bound proteins with a major role in the creation and amplification of the ROS signal in a variety of plant immune and developmental responses. RBOHs are responsible for releasing O_2_^-^ into the apoplast and are regulated by Ca^2+^ through EF-hand motifs in the *N* terminus (Baxter et al., 2014). This extracellular ROS activity is thought to be responsible for the activation and amplification of a self-propagating ROS wave, thus explaining the systemic nature of the immune response (Mittler et al., 2011). Given the role of Ca^2+^ in activating RBOHs, calcium-dependent protein kinases (CDPKs) are another possible node of convergence in ROS signaling. Tobacco leaves infiltrated with a constitutively active version of NtCDPK2, usually only activated by pathogen attack, responded to mild abiotic stress with an expanding HR (Kobayashi et al., 2007). Additionally, plasma membrane Ca^2+^ transporters were shown to mediate tolerance to different oxidative stresses in virus-inoculated tobacco (Shabala et al., 2011).

## ROLE OF ROS IN SYSTEMIC RESPONSES AND PROGRAMMED CELL DEATH

Systemic resistance primes plant-wide defense responses following an initial localized trigger. Systemic acquired resistance (SAR) refers to the enhanced response observed after pathogen attack, systemic acquired acclimation (SAA) to a similar response elicited from non-lethal doses of abiotic stressors, and induced systemic resistance (ISR) to the response to symbiotic associations with mutualistic microbes. Systemic responses to wounding and herbivory have also been described (Baxter et al., 2014). While these responses have distinct molecular signatures, they all involve ROS signaling. Comparison of *Arabidopsis* transcriptome responses to a variety of stressors (MV, O_3_, fungal toxin, 3-aminotriazole herbicide, and antioxidant-compromised mutants) identified a core set of ROS-dependent response genes (Gadjev, 2006).

Treatment with both O_3_ and fungal elicitors activates an oxidative burst response and components of the HR signaling pathway (Sandermann, 1996; Wohlgemuth et al., 2002). The *rcd1* mutant in *Arabidopsis* displays both high sensitivity to O_3_ and a deficient programmed cell death (PCD) response, which is known to be regulated by ROS (De Pinto et al., 2012). Transcriptome analysis of the *rcd1* mutant found clusters of O_3_-upregulated genes with enrichment of GO terms “response to salicylic acid stimulus” and “response to bacterium.” In addition WRKY70 and SGT1b, required for ubiquitin mediated protein degradation, were found to be involved in regulation of ROS-induced PCD (Brosché et al., 2014).

## CROSS-TOLERANCE MAY RESULT FROM ROS SIGNAL OVERLAP

**FIGURE 1:**
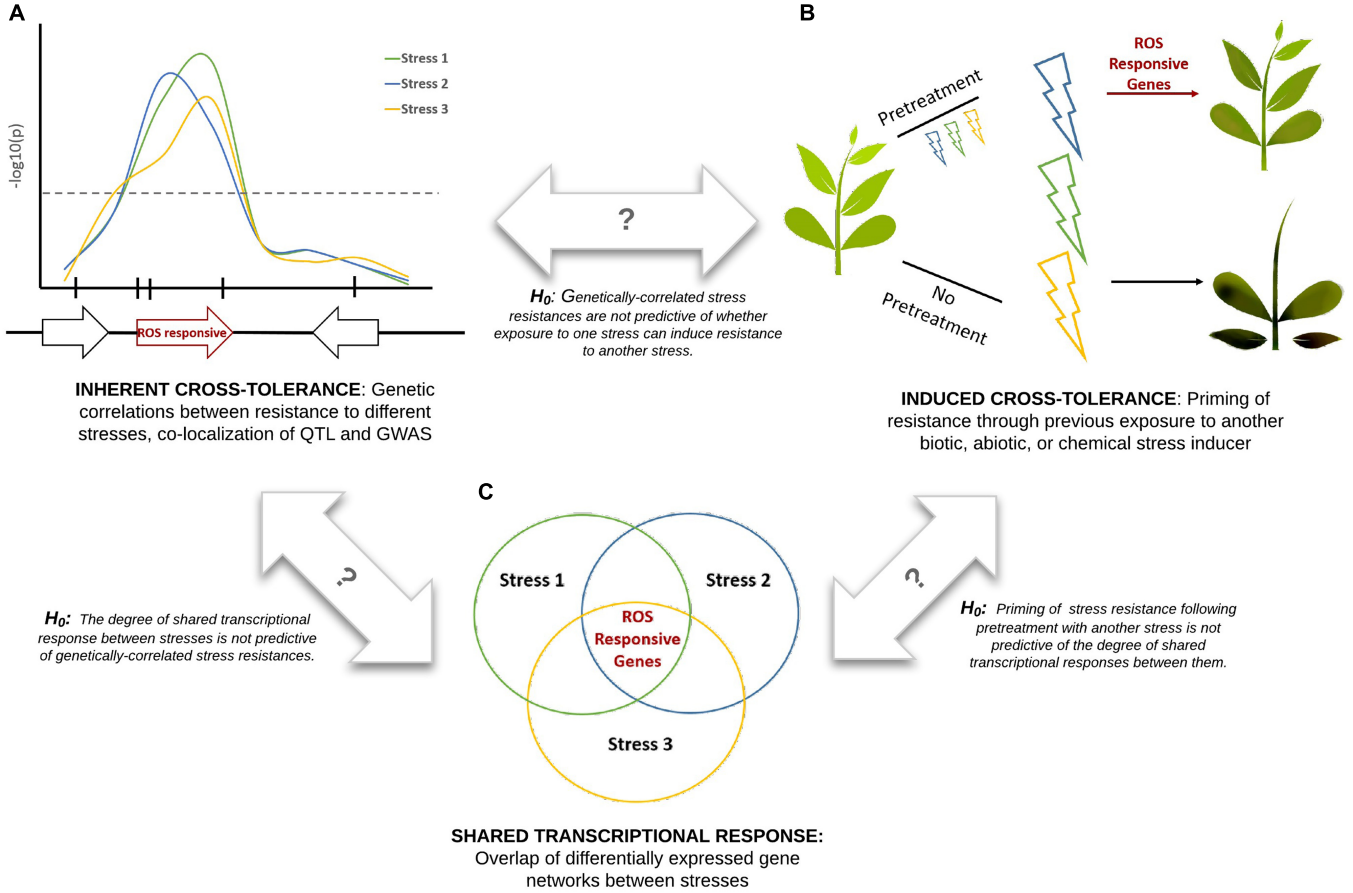
**Integration of experimental approaches to studying plant cross-tolerance.** Three hypothetical stresses (biotic or abiotic) are shown in blue, green, and yellow. Marker-trait association studies **(A)**, pretreatment effect studies **(B)**, and gene expression studies **(C)**, when performed under consistent experimental conditions, can be used to test a series of null hypotheses. ROS signaling is proposed to play a key role in cross-tolerance responses.

Cross-tolerance, also referred to as cross-resistance or cross-protection (Tippmann et al., 2006), refers to the enhanced ability of a plant to tolerate multiple stresses. Here we attempt to unify three distinct types of plant stress tolerance studies (**Figure 1**): datasets describing transcriptional overlap between stress responses; datasets describing the priming or suppression of plant stress responses following exposure to a different type of stress (induced cross-tolerance); and datasets describing genetic overlap between resistance to different stresses (inherent cross-tolerance). Comparison of these datasets within a single experimental system would create a powerful platform for understanding systemic resistance and would allow a series of null hypotheses to be tested (**Figure 1**). Correlations between these datasets may be due in part to common ROS signals that are refined to provide stress-specific information through differences in localization, amplitude, and frequency of the ROS electrical wave (Mittler et al., 2011).

## INDUCED CROSS-TOLERANCE

Following the recognition of systemic resistance in plants, agronomists have pursued the possibility of inducing systemic resistance in crops through chemical means. A growing family of agrochemical products targeted as “defense-inducers” aims to emulate or induce the accumulation of H_2_O_2_ in SAR [thoroughly reviewed by (Gozzo and Faoro, 2013)]. Exogenously applied salicylic acid has been used to attempt to improve plant responses to drought (Alam et al., 2013), salt stress (Hasanuzzaman et al., 2014), iron-deficiency (Kong et al., 2014), pesticides (Singh et al., 2013), and heavy metals (Siddiqui et al., 2013). Some fungicides and herbicides appear to have fortuitous secondary uses as defense inducers. In field trials against soybean stem rot, early application of the diphenyl ether herbicide Lactofen was found to significantly reduce disease severity in a high disease-pressure environment (Dann et al., 1999), possibly due to the induced overexpression of PR proteins and thaumatin/osmotin-like proteins also involved in osmotic stress tolerance (Graham, 2005). Treatment of tobacco with the fungicide pyraclostrobin enhanced resistance to both tobacco mosaic virus and *Pseudomonas spp.* infection, even in SA-deficient mutants, suggesting a SA-independent response (Herms et al., 2002). Dual application of the fungicide chlorothalonil and the antiozonant compound EDU resulted in higher resistance to O_3_ injury and elevated levels of glutathione (Hassan, 2006). Pretreatment of potato roots with aluminum (Al^3+^) resulted in fourfold higher leaf resistance to late blight *(Phytophthora infestans)* and the accumulation of SA and distal NO (Arasimowicz-Jelonek et al., 2013). The common theme in these responses seems to be an initial disruption of cellular ROS homeostasis by the inducer, followed by enhanced detoxification capacity. The same principle is employed by herbicide safeners, which are chemical seed treatments that induce expression of detoxification enzymes (GSTs and cytochrome P450s) in the germinating seedling, protecting it from subsequent herbicide injury. The exact mechanisms through which safeners confer herbicide resistance are unknown, although oxidized lipids (“oxylipins”) have been proposed to play a key role (Riechers et al., 2010). A clear link between oxylipins and systemic resistance was demonstrated using the *lipoxygenase3* (*lox3*) mutant of maize, which is deficient for 9-oxylipin production in roots and shows enhanced resistance to fungal diseases and constitutive ISR response (Constantino et al., 2013).

Direct application of ROS by oxidative agents has also been investigated as a potential means of inducing cross-tolerance. H_2_O_2_ is a known activator of antioxidant defenses and has been used as seed pretreatment for wheat planted into environments that experience drought and/or salt stress (Wahid et al., 2007; He and Gao, 2009). O_3_ is an attractive inducer because doses can be carefully calibrated for low toxicity. Inoculation of tobacco plants with either O_3_ or UV-light resulted in accumulation of SA and PR proteins and induced resistance to tobacco mosaic virus (Yalpani et al., 1994). Tomato plants showed increased resistance to cucumber mosaic virus after being regenerated from calli previously treated with a low dosage of O_3_ (Sudhakar et al., 2007), suggesting that the memory of oxidative stress persists through ontogeny.

The success of inducers has been varied, possibly due to limited understanding of the dynamics between triggers and resistant responses. For example, bean plants pretreated with BTH, a functional analog of SA, were more sensitive to O_3_ fumigation 1–2 days after BTH pre-treatment but more resistant to O_3_ fumigation 5–7 days after BTH pre-treatment (Iriti et al., 2003). Many other studies similarly highlight the importance of dosage and timing to the success of an inducer (Sandermann, 1996; Rao and Davis, 1999; Geetha and Shetty, 2002; Buschmann et al., 2005; Herman et al., 2008; Hayat et al., 2010).

## INHERENT CROSS-TOLERANCE

Development of crop varieties with durable resistance to multiple stresses is a nearly universal goal in plant breeding. Qualitative, race-specific disease resistance can be quickly overcome because of the selective pressure it imposes on pathogen populations. Quantitative, broad-spectrum disease resistance (QDR) involves multiple loci encoding resistance alleles, usually of modest effect, that provide a more durable resistance against multiple races and species of pathogen. QDR has also been shown to improve the durability of qualitative resistance (Brun et al., 2010). Empirical evidence for QDR arises from QTL meta-analysis showing clustering of QTL for different diseases (Wisser et al., 2005), and from high genetic correlations between resistances to different diseases observed across collections of genetically diverse individuals (Wisser et al., 2011). The enhanced capacity of specific individuals within a species to resist multiple stresses could result from a variety of mechanisms, including plant and cell architecture, signal transduction, and the detoxification response (Poland et al., 2009). However, inherent cross-tolerance between widely divergent stresses suggests a basal generalized ability to avoid cellular redox disequilibrium.

A plant’s ability to tolerate exogenously applied ROS has been used as a simple initial screen for multiple stress resistance. A survey of diverse *Arabidopsis* accessions found great natural variation in O_3_ tolerance (Brosché et al., 2010). Shared resistance to O_3_, SO_3_ and MV was found between tolerant *Lolium perenne* and tobacco biotypes (Shaaltiel et al., 1988). A global survey of *Medicago truncatula* accessions found a positive correlation between tolerance to oxidative stress and drought stress, as well as a significant negative correlation between basal ROS level and oxidative stress tolerance (Puckette et al., 2007). Tolerances to oxidative stress and drought stress in a set of rice landraces and improved varieties were found to be strongly correlated both with each other and with population structure, with *japonica* lines and improved varieties showing higher tolerance to both stresses than *indica* lines (Iseki et al., 2013). A positive correlation between oxidative stress tolerance, antioxidant activity and drought tolerance was found in a set of maize inbred lines evaluated for resistance to MV, acifluorfen herbicide and SO_2_ (Malan et al., 1990). MV-based screening of progeny from a cross between a drought-tolerant and a drought-sensitive rice variety was used to predict drought tolerance in a dry-season rice breeding program (Iseki et al., 2014).

Glutathione-S-transferase (GST) genes play an important role in the maintenance of ROS equilibrium and have been widely reported to play a role in biotic and abiotic stress resistance (Wagner et al., 2002; Dean et al., 2005). Comparison of MV-induced stress transcriptomes between *japonica* and *indica* rice subspecies indicated GST activity as a major differentiated GO module (Liu et al., 2010). A multivariate GWAS to identify sources of multiple disease resistance in maize pinpointed a GST gene associated with resistance to three major fungal diseases (Wisser et al., 2011). In screens for resistance to specific diseases in the high resolution nested association mapping population of maize, GSTs, cytochrome P450s, and receptor-like kinases (RLKs) were identified as candidates (Kump et al., 2011). A meta-analysis of published QTL studies in rice for resistance to several diseases yielded a type III GST as a candidate gene for functional studies (Wisser et al., 2005). It is not clear whether GSTs are acting directly on specific, pathogen-derived toxins or indirectly on ROS-derived compounds, like peroxidized lipids, that result from stress.

## MODEL TO CROP

Translational genomics relies on conservation of gene function and network architecture between model and crop species, and the likelihood of success is expected to depend on the model-crop divergence time. However, networks of co-expressed genes are more stable across evolutionary time than individual genes. For example, a study comparing abiotic stress-responsive gene networks between *Arabidopsis* and Medicago found that ~60% of *Arabidopsis* genes could be assigned a clear ortholog in Medicago despite a divergence time of ~125 million years (Hyung et al., 2014). Construction of weighted co-expression gene networks in *Arabidopsis* and rice under bacterial and drought stress identified a number of “response to oxidative stress”-annotated modules that were upregulated in both species in response to both stresses (Shaik and Ramakrishna, 2013), suggesting that with appropriate methodology, biological inferences can be translated between distantly related genomes. Another hurdle in translational genomics is the transition from greenhouse or growth chamber to a field environment. Stress factors in controlled environments are usually imposed in isolation, whereas crop plants under field conditions are nearly always exposed to multiple stresses that may interact with each other (Mittler, 2006). Sudden imposition of stress in controlled environments also downplays the importance of plant acclimation and the mild, chronic stresses that prevail in nature. Part of the solution may involve the adoption of new technologies that allow increased experimental control over field environments. For example, rainout shelters allow manipulation of soil water profile and drought stress in a field setting, and Free Air Concentration Enrichment (FACE) facilities alter atmospheric variables, including CO_2_ and O_3_ concentrations, to obtain realistic estimates of their effects on crop yields (Ort et al., 2006). Plant resistance to multiple stresses may be constrained by antagonistic interactions. However, evidence from transcriptional, quantitative genetic, and agronomic datasets shows that plants respond to multiple biotic and abiotic stresses using similar, ROS-mediated mechanisms. Deliberate integration of information between these different types of datasets will help us understand and exploit cross-tolerance, providing improved agronomic and genetic tools for a more sustainable, climate-resilient agriculture.

## Conflict of Interest Statement

The authors declare that the research was conducted in the absence of any commercial or financial relationships that could be construed as a potential conflict of interest.
